# Pharmacy in South America

**Published:** 1864-02

**Authors:** 


*Pharmacy in South America.*—The Pharmaceutical Association of Buenos Ayres has lately received the official recognition of the Argentine Government. It has also petitioned the Government for the formation of a School of Pharmacy at Buenos Ayres. The Society pledges itself to maintain an official journal of the Society. The journal is in the Spanish language and is published quarterly.

